# Plant microRNAs and their role in defense against viruses: a bioinformatics approach

**DOI:** 10.1186/1471-2229-10-138

**Published:** 2010-07-01

**Authors:** Álvaro L Pérez-Quintero, Rafik Neme, Andrés Zapata, Camilo López

**Affiliations:** 1 Universidad Nacional de Colombia, Bogotá, Departamento de Biología, Oficina 222. Calle 45 Cra 30. Bogota D.C. Colombia

## Abstract

**Background:**

microRNAs (miRNAs) are non-coding short RNAs that regulate gene expression in eukaryotes by translational inhibition or cleavage of complementary mRNAs. In plants, miRNAs are known to target mostly transcription factors and are implicated in diverse aspects of plant growth and development. A role has been suggested for the miRNA pathway in antiviral defense in plants. In this work, a bioinformatics approach was taken to test whether plant miRNAs from six species could have antiviral activity by targeting the genomes of plant infecting viruses.

**Results:**

All plants showed a repertoire of miRNAs with potential for targeting viral genomes. The viruses were targeted by abundant and conserved miRNA families in regions coding for cylindrical inclusion proteins, capsid proteins, and nuclear inclusion body proteins. The parameters for our predicted miRNA:target pairings in the viral genomes were similar to those for validated targets in the plant genomes, indicating that our predicted pairings might behave *in-vivo *as natural miRNa-target pairings. Our screening was compared with negative controls comprising randomly generated miRNAs, animal miRNAs, and genomes of animal-infecting viruses. We found that plant miRNAs target plant viruses more efficiently than any other sequences, but also, miRNAs can either preferentially target plant-infecting viruses or target any virus without preference.

**Conclusions:**

Our results show a strong potential for antiviral activity of plant miRNAs and suggest that the miRNA pathway may be a support mechanism to the siRNA pathway in antiviral defense.

## Background

RNA silencing is a conserved defense mechanism that plants and other eukaryotes use to protect their genomes against aberrant nucleic acids. This process uses short RNAs (20-30 nt) to recognize and manipulate complementary nucleic acids [1,2]. At least five classes of these small regulatory RNAs have been characterized, including microRNAs (miRNAs), small interference RNAs (siRNAs), transacting siRNAs (ta-siRNAs), natural antisense siRNAs (nat-siRNAs) and, in metazoans, the Piwi-interacting RNAs [3,4]. miRNAs and siRNAs are chemically indistinguishable and participate in partially overlapping pathways; both are derived from double-stranded RNA (dsRNA) and are then processed into 21-22 nt single stranded molecules by Dicer or a Dicer-like enzyme; later, they are incorporated into the RNA-induced silencing complex (RISC) to guide the cleavage or translational repression of the complementary strand [1,5]. The main differences between miRNAs and siRNAs lie in their biogenesis and in their target molecules. siRNAs are generally derived from endogenous aberrant dsRNAs or from exogenous agents such as viruses, and silence the same molecule from which they originated. miRNAs, instead, originate from nuclear genes and act *in trans*, silencing mRNAs from other genes [6,7].

In plants miRNAs were described first in *Arabidopsis *[8,9], and later in other species. To date, there over 2200 plant miRNAs from over 30 species available at the miRBase [10]. Most of these miRNAs target transcription factors and thus are implicated in diverse aspects of plant growth and development [11,12].

In addition to regulate the endogenous expression of some genes, miRNAs could have a direct role in viral defense. This has been shown for various cases in animal-infecting viruses. For example miR-32 restricts the replication of the primate foamy virus type 1, miR-122 targets the hepatitis C virus and at least four miRNAs expressed in T-cells impair HIV replication [2,13-15]. Also an important role for miRNAs in antiviral defense in humans has been suggested through bioinformatics [16]. Likewise, animal-infecting viruses can encode miRNAs to regulate both the viral life cycle and the interaction between viruses and their hosts [17,18].

Whereas siRNAs are known to play an important and direct role in antiviral defense in plants [19,20], so far, there has not been proof of naturally occurring plant microRNAs with antiviral activity. It has been shown, using genetically modified viruses and plants, that complementarity between a plant miRNA and a virus genome is enough for antiviral activity. Transgenic tobacco and *Arabidopsis *plants displayed resistance against *Cucumber mosaic virus *(CMV), *Turnip yellow mosaic virus *(TYMV) and *Turnip mosaic virus *(TuMV) when expressing artificial miRNAS directed against regions in the viruses' genomes [21-23]. Also, inserting the target sequence of host plant's miRNAs in the virus genome can impair virus infectivity; however, the virus can escape rapidly of the miRNA action by mutations [24].

It has been suggested that virtually any endogenous small RNA could hold an intrinsic, albeit fortuitous, antiviral potential (by random complementarity) that is independent of its cellular function [15,24-26]. Also, several sequences of 20-25 nt located within *Arabidopsis *intergenic regions share perfect or near perfect complementarity with a variety of plant virus genomes, but have not been validated as miRNAs yet [27]. There are also a large number of non-conserved RNAs with unknown targets ("orphan" miRNAs) that could have an antiviral role and constitute a reservoir of defensive molecules due to their complementarity to invading viral genomes [25].

In this work, we present a bioinformatics approach to explore the possibility of endogenous plant miRNAs having a role in antiviral defense by targeting the genomes of plant-infecting viruses and the results are considered in the context of the evolution of plant-virus interactions.

## Results

The set of plant miRNAs (n = 911) from six plants was screened for targets against a set of genomes of plant infecting viruses (n = 119) resulting in several putative targets (any miRNA-target pair predicted by miRanda is considered a hit). The plant with most hits was *O. sativa *with 165, which was expected since most of the miRNAs in the dataset belong to this species (353). The matching percentage, which relates the number of hits to the sample size (miRNAs × viruses genomes), was similar for all species, around 0.2%. The plant with the highest matching percentage (0.2813%) was *Z. mays*, and the lowest was *A. thaliana *(0.1579%). Overall out of the 911 plant miRNAs used in the screenings, 267 (28%) had targets in the genomes of plant viruses; we name these "positive miRNAs". The percentage of positive miRNAs was different for each plant, being lowest (22%) in *A. thaliana *and highest (43%) in *Z. mays*. The percentage of "positive viruses" (viruses that were targeted by at least one miRNA) was lowest for *S. bicolor *(34%) and highest for *A. thaliana *(80%) (Table 1). Thus every plant has a different repertoire of miRNAs with a potential capacity of targeting viruses.

**Table 1:** Statistics for plant microRNAs vs plant viruses' genomes screenings

	*A. thaliana*	*G. max*	*O. sativa*	*S. bicolor*	*V. vinifera*	*Z. mays*
**Number of miRNAs**	187 miRNAs	69 miRNAs	353 miRNAs	72 miRNAs	140 miRNAs	90 miRNAs

**Number of viruses (total size)**	20 (149. 9 Kb)	20 (146. 1 Kb)	20 (223.7 Kb)	20 (166. 2 Kb)	16 (166. 4)	23 (188. 8 Kb)

**Hits^a^**	49	21	165	27	59	51

**Matching percentage (Hits/Sample size) × 100**	0.1579	0.2053	0.1972	0.2265	0.2558	0.2813

**Positive miRNAs^b ^(%)**	41 (22%)	18 (26%)	103 (29%)	25 (34%)	41 (29%)	39 (43%)

**Positive viruses^c ^(%)**	16 (80%)	10 (50%)	15 (75%)	6 (30%)	10 (50%)	9 (45%)

a) MiRanda predicted microRNA:target pairs, b) microRNAs with possible targets in viruses genomes, c) viruses with miRNAs targets in their genomes.

**Figure 1:**
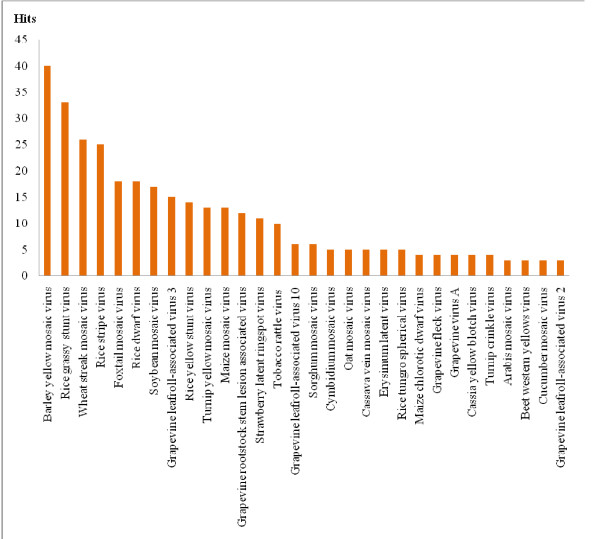
**Plant infecting viruses targeted by plant miRNAs**. Only viruses with more than 3 hits are shown.

In total, 51 of the 74 (69%) viruses screened were "positive viruses", thus not all plant-infecting viruses can be targeted by their host's miRNAs. Some viruses were highly targeted by plant miRNAs. For example, the Barley Yellow Mosaic Virus, to which both *O.sativa *and *Z.mays *are unsusceptible, displayed the highest number of miRNA targets (Figure 1) [28].

**Figure 2:**
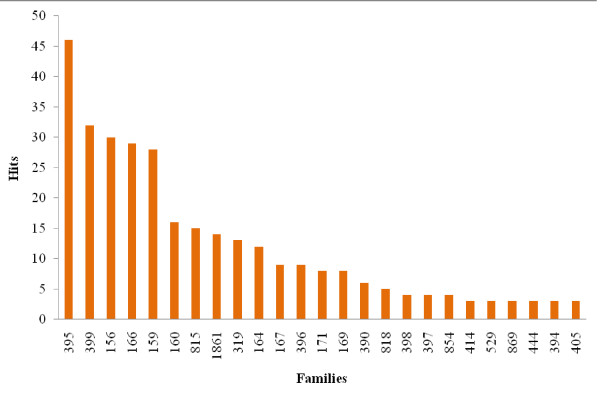
**miRNAs families and the number of putative targets in the genomes of plant viruses**. Only families with more than 3 hits are shown.

miRNAs can be grouped according to sequence similarity in families. In total 233 miRNA families were screened against the viral genomes and 74 families (32%) resulted in positive targets. Families that are relatively well conserved across the plant kingdom and have multiple copies in the genome were particularly successful in producing hits; this may be a consequence of this families being overrepresented in every screening (Figure 2). Families 156, 395, 159, 166, 160 which are present in at least five of the six plant species and are encoded by at least two loci in each plant genome were among the ones with more potential targets. Some families with unknown or non-validated targets (i.e. 495, 414, 815, 818, 854, 529, and 1861) also produced multiple, yet fewer, hits in the viruses' genomes. These results suggest that abundant and conserved plant miRNA families potentially target viruses.

To validate our hypotheses that plant-infecting viruses are more likely to be targeted by plant miRNAs than by other sequences and that plant miRNAs preferentially target plant-infecting viruses over other sequences, we conducted the following analyses. We created a group of negative controls to screen for miRNA targets in the following cases *i*) animal miRNAs vs plant virus genomes, *ii*) random generated miRNAs vs plant virus genomes, *iii*) randomized plant miRNAs vs plant virus genomes and *iv*) plant miRNAs vs animal virus genomes. As a positive control we screened the set of plant miRNAs against their validated target sequences.

The screenings were compared using four miRanda parameters: the free-folding energy of the miRNA:target pair, the identity, the Z-score and the miRANDA score. All putative targets in each screening had high identity percentage (min 58%), high Z-score (min 6.8) and highly negative free-folding energy (maximum -23 kcal/mol) (Table 2). No statistically significant differences were found between the different screenings for these three parameters, indicating that all the alignments found are very similar and therefore comparable. Since there are no differences between the positive control screening and all the others, we can conclude that our positive miRNAs are pairing with their targets as well as some plant miRNAs pair with their known and validated targets in the plant genomes.

**Table 2:** miRANDA scoring values for each screening

			Negative controls				Positive controls
	**Screenings**	**Plant miRNAs vs Plant viruses**	**Animal miRNAs vs Plant viruses**	**Rand1 vs Plant viruses**	**Rand2 vs Plant viruses**	**Plant miRNAs vs Animal viruses**	**Plant miRNAs vs Control Targets**

Free-folding Energy (kcal/mol)	min	-23.05	-23.01	-23.01	-23.02	-23.03	-23.01
	
	mean	-26.34	-27.22	-26.89	-26.07	-26.16	-28.5
	
	max	-43.99	-45.13	-39.87	-38. 88.88	-36.26	-45.25
	
	*p value*		*0.290*	*0.091*	*0.184*	*0.173*	*0.0626*

Identity (%)	min	0.66	0.58	0.68	0.64	0.61	0.7059
	
	mean	0.78	0.78	0.79	0.78	0.79	0.814
	
	max	0.94	1	0.94	0.94	0.94	1
	
	*p value*		0.784	0.212	0.548	0.323	0.0592

miRANDA score	min	114	96	107	87	109	126
	
	mean	158.8	142	147.8	142.5	142.4	167.9
	
	max	182	187	182	180	182	194
	
	*p value*		↓* <***0.001***	*0.548*	↓* <***0.001***	↓* <***0.001***	↑* <***0.001***

Z score	min	7.009	6.834	7.009	6.995	7. 102	7.200
	
	mean	8.815	8.003	7.967	8.004	8.413	9.001
	
	max	10.080	9.895	8.967	9.746	10.426	11.581
	
	*p value*		0.643	0.342	0.423	0.262	0.089

p values are the result of pairwise Wilcoxon test between each screening and the plant microRNAs vs Plant viruses screening, * significant differences α = 0.05, ↓ significantly lower, ↑ significantly higher

The miRanda score of the positive control was significantly higher than the score of the plant miRNA vs plant viruses screening, while the miRanda score for three of the four negative controls was significantly lower. However, all miRANDA scores are above the threshold of what is considered necessary for biological activity. We should also take into account that this parameter gives a high weight to pairing in the 5'region of the miRNA which is not as crucial for plant miRNAs activity as for animal miRNAs (Table 2) [29].

**Figure 3:**
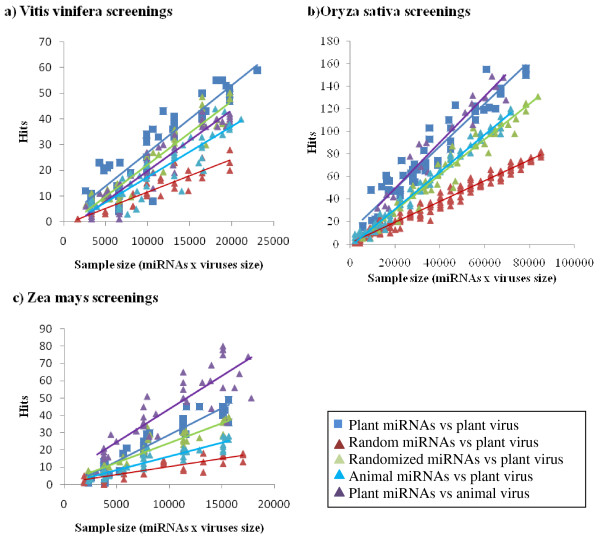
**Hits for screenings with different sample sizes subsets**. A) Screenings for *Vitis vinifera*,. B) Screenings for *Oryza sativa*. C) Screening for *Zea mays*

Next, our screening was compared with the negative controls using the matching percentage. To discard errors due to sample size effect, various data subsets with different sample sizes of miRNAs and viral genomes were randomly generated, screened again and then averaged (Table 3). The matching percentages for plants miRNAs to plant viruses were significantly higher than to animal miRNAs and the two types of random miRNAs. This indicates that the plant viruses might be preferentially targeted by plant miRNAs than by other sequences. On the other hand, comparisons of the matching percentages for plant miRNAs to plant and animal viral genomes did not show a clear trend (Table 3). For example, the miRNAs of *V. vinifera *seem to preferentially target plant viruses than animal viruses (Figure 3A) while the opposite was the case for *A. thaliana, S. bicolor *and *Z. mays *(Figure 3B). And, the miRNAs from *O. sativa *and *G. max *showed similar preference for the genomes of both plant and animal viruses (Figure 3C). No clear conclusion can then be drawn as to the specificity of plant miRNAs for plant viruses.

**Table 3:** Matching percentages of plant microRNAs vs plant viruses screenings compared to negative controls

			Negative controls			
**Plant microRNAs vs Plant viruses screenings**			**Animal microRNAs vs Plant viruses**	**Rand1 vs Plant viruses**	**Rand2 vs Plant viruses**	**Plant microRNAs vs Animal viruses**

*A. thaliana*- m vs *A. thaliana *- viruses	mean	0.16812	0.14473	0.08857	0.12714	0.2130
	
	*p value*		↓ **< 0.0001**	↓ **< 0.0001**	↓ **< 0.0001**	↑ **< 0.0001**

*G. max *microRNAs vs *G.max *- viruses	mean	0.2088	0.10584	0.09664	0.11972	0.18624
	
	*p value*		↓ **< 0.0001**	↓ **< 0.0001**	↓ **< 0.0001**	*0.2394*

*O. sativa *microRNAs vs *O.sativa *viruses	mean	0.2321	0.16285	0.09647	0.1481	0.2295
	
	*p value*		↓ **< 0.0001**	↓ **< 0.0001**	↓ **< 0.0001**	*0.3983*

*S. bicolor *microRNAs vs *S.bicolor *- viruses	mean	0.23237	0.14577	0.10179	0.12557	0.3656
	
	*p value*		↓ **< 0.0001**	↓ **< 0.0001**	↓ **< 0.0001**	↑ **< 0.0001**

*V.vinifera *microRNAs *V.vinifera *viruses	mean	0.2741	0.17348	0.10818	0.18675	0.22115
	
	*p value*		↓ **< 0.0001**	↓ **< 0.0001**	↓ **< 0.0001**	↓ **< 0.0001**

*Z. mays *microRNAs vs *Z. mays *viruses	mean	0.26809	0.16263	0.10903	0.2446	0.4445
	
	*p value*		↓ **< 0.0001**	↓ **< 0.0001**	↓ **< 0.0001**	↑ **< 0.0001**

p values are the result of pairwise Wilcoxon test between each screening and the plant microRNAs vs Plant viruses screening, α = 0.05, ↓ significantly lower, ↑ significantly higher

**Figure 4:**
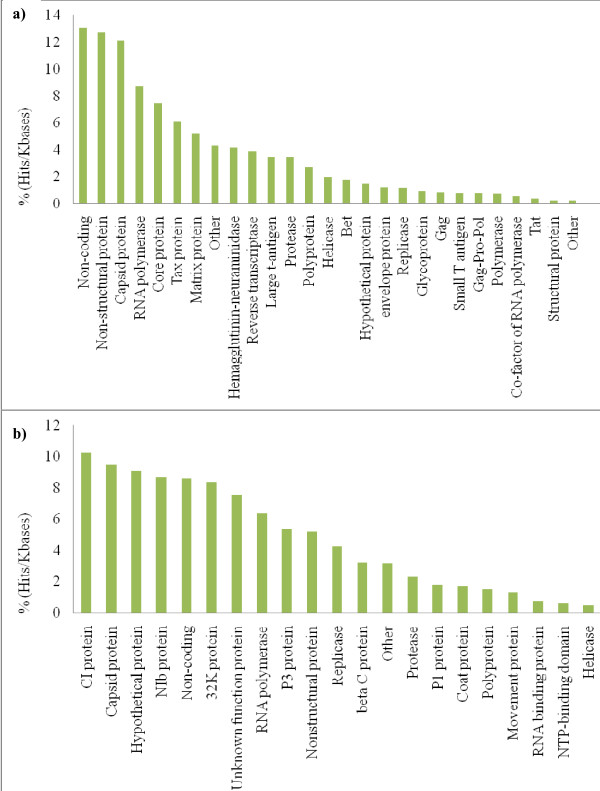
**Viruses' regions targeted by microRNA**. Bars show the number of hits for each region divided by the average size in kbs of that region in the various genomes. Percentages are given over the total hits/kbs. a) Plant virus regions targeted by plant miRNAs, b) Animal virus regions targeted by plant miRNAs

The genomes of plant viruses were targeted in multiple regions by several plant miRNAs. The most targeted regions were those coding for RNA polymerases, cylindrical inclusion (CI) proteins, capsid proteins and nuclear inclusion body (Nib) proteins (Figure 4A). Silencing in any of these regions is likely to impair virus replication. Plant miRNAs also target most frequently the RNA polymerase genes in animal viruses (Figure 4B). However, there is a stronger preference to target coding sequences in plant viruses than in animal viruses. Therefore, plant miRNAs seem to be more directed to impair the fitness of plant viruses.

## Discussion

Using a bioinformatics approach we found that plant miRNAs potentially target genomic regions in plant-infecting viruses. To validate our results we carried out several positive and negative controls and these showed that the genomes of plant viruses are preferentially targeted by their host's miRNAs but were not conclusive regarding the specificity of plant miRNAs for the genomes of plant viruses. A similar trend has been found using a bioinformatics approach with animal miRNAs vs animal viruses [16], where the miRNA pathway has been proved to have antiviral role in Metazoans [2,13-15]. This suggests that our predicted pairings could also have a biological function, although an experimental biological validation is necessary. It is possible that some of the viral targets found in this study are the result of purely fortuitous matches as has been suggested by various authors [15,26,27,30]. Even if these pairings are the result of chance instead of selection, it is possible that given the right physiological circumstances (e.g. high expression of the miRNAs, lack of silencing suppressor in the virus) these miRNAs would efficiently silence the predicted targets. This hypothesis is supported by studies showing that artificial miRNAs can mediate antiviral defense in plants and that complementarity with the target is enough to produce resistance [21-24]. Also, plants defective in miRNA-silencing have shown to be more susceptible to some viruses [31].

It was reported that human miRNAs were more likely to target the genomes of human-infecting viruses over non-host's viruses [16]. Such specificity could not be demonstrated for plant miRNAs in the present study. However, a large amount of the targets we found for plant miRNAs in the genomes of animal viruses are in non-coding regions and are therefore unlikely to impair viral activity (Figure 4). Additionally, some predicted targets of plant miRNAs were found both in plant and animal viruses (e.g. capsid genes) which may indicate a preference to target conserved regions in viruses. Finally, it is possible that the genomes of plant-infecting viruses are undergoing rapid evolution to avoid targeting by plant miRNAs, therefore giving lower matching percentages than expected. This is plausible since it has been shown that viruses can rapidly evolve to escape miRNA targeting in plants [24].

To identify possible plant miRNAs in the viruses genomes we used strict parameters based on experimentally valid miRNA:target pairings to ensure potential biological activity. Even considering the inherent difficulties of the computational prediction of miRNA targets, which often results large number of false positive targets [32], it is possible that our conservative approach has underestimated the number of candidate targets. Increasing evidence has shown that miRNA-mediated silencing in plants can occur in relaxed miRNA:target pairings, mainly leading to translational arrest instead of mRNA cleavage, although the mechanisms are not fully understood [17,33-36]. Once the criteria for miRNA-mediated translational arrest in plants are fully understood, new approaches searching for plant miRNA targets in viral genomes may be necessary.

We found that miRNAs from deeply conserved and highly expressed families (e. g. families 156, 395, 159, 166, 160) have more potential targets in the viruses' genomes. This could suggest a way in which abundant plant miRNAs are selected to have multiplicity of functions including pathogen defense. This is supported by the fact that these families have multiple targets within the plant genomes [33], and some of them have been shown to be differentially expressed in response to stresses. For example, miRNAs 395 and 399 are responsive to abiotic stress (phosphorus and sulfates starvation) [37,38], and miRNAs 156, 159 and 160 are responsive to viral infections [39-42].

By contrast, the more phylogenetically restricted families (e.g. families 495, 414, 818, 854, 1861), may be participating in more specific plant-virus interactions. Indeed, in some plants there is a large diversity of non-conserved and "young" miRNAs with still unknown targets that could be potentially employed against viral sequences [43,44]. The lack of potential antiviral activity for some microRNA families could also be the result of them being expressed at very low levels or in a tissue or cell-specific manner, thus being less likely to play a significant role in antiviral defense.

It is also important to consider some arguments that do not support a putative function of plant miRNAs as an effective option for antiviral defense. First, most viruses encode for silencing repressors, which could directly interfere with the miRNA machinery [27,45,46]. Second, viral genomes evolve much faster than host miRNAs [11,24]. Third, the miRNA signal is neither systemic nor quickly amplified [26]. Nevertheless, using miRNAs to protect against virus might be an advantageous preemptive measure (a plant would be resistant to viruses that has never encountered before) benefitting of their ability to pair with multiple targets [26].

The apparent inadequacy of miRNAs as an antiviral defense mechanism may indicate that their role is not as direct as siRNAs. On one side miRNAs may simply act as a support mechanism for siRNAs. On the other side, the targets found here may be a reflection of a virus adaptation phenomenon in which they take advantage of the host miRNAs to suppress their own replication to evade immune elimination and establish in this way a persistent infection as has been suggested by Mahajan *et al*., [47]. In this case the role of miRNAs would be to reach an equilibrated host-virus interaction [47].

Also, these results can be discussed in the context of the hypothesis proposed by Lu *et al*., [26], which states that early in plant evolution miRNAs played an important role in anti-viral defense and then novel functions evolved after the requirements of survival were satisfied [26,33]. At this initial time, plant miRNAs may have been crucial for shaping the host ranges of several virus groups. Then, some of these "antiviral miRNAs" might have been selected to regulate endogenous genes after fortuitous matching. Both the rapid evolution of viruses and the necessity of precise gene regulation could have worked as selective pressures towards the modern miRNA pathway since the requirement for a high degree of complementarity between plant miRNAs and their targets can act as a stabilizer, preventing sequence drift even over long periods of evolutionary time [43]. Many miRNAs might have been originated from invading viral sequences, a pathway for miRNA evolution that has been suggested previously for plants [48]. Additionally, bioinformatics evidence suggests a transition from viral sequence to siRNA to miRNA gene in plants [49]. Our candidate targets may be an indication of these virus-derived miRNAs, especially those found for phylogenetically restricted miRNA families with unknown genomic targets.

## Conclusions

Our work presents initial evidence for the suspected potential of antiviral activity mediated by plant miRNAs, which is likely to have played a role in early plant evolution and in shaping host ranges for plant infecting viruses.

## Methods

### Dataset

miRNA sequences from six plants (*Arabidopsis thaliana, Glycine max, Oryza sativa, Sorghum bicolor, Vitis vinifera*, and *Zea mays*) were downloaded from the miRBASE [10]. These species were selected for having at least 60 available sequenced miRNAs as late as March 2009, and for being hosts of at least 10 plant-infecting viruses with fully sequenced genomes. For comparisons, miRNAs from eight Metazoan species (*Coenorhabditis elegans, Drosophila melanogaster, Dario rerio, Gallus gallus, Homo sapiens, Mus musculus, Ornithorhynchus anatinus*, and *Pan troglodytes*) were selected and 50 miRNAs sequences for each animal were also downloaded from the miRBASE [10].

Complete genome sequences for plant and animal-infecting viruses were obtained from Genbank [50]. Host ranges and related information for plant viruses were consulted using the Description of Plant Virus Database, DPVWeb [51] and the Plant virus Database, VIDE [52]. For animal-infecting viruses we used the International Committee on Taxonomy of Viruses (ICTV) database [28].

Two sets of random miRNAS were made, one using a Perl script generating random 21-nucleotide sequences [53], and another one by randomizing the plant miRNA sequences with the Bioedit software [54], doing 1000 random swap operations.

Information for verified targets of plant miRNAs were obtained from Tarbase [55], the *Arabidopsis thaliana *small RNA project [56], the *Arabidospsis *MPSS Database [57], the Plant microRNA Database [58], or primary literature [11,59,60]. The corresponding sequences were downloaded from Genbank [50].

### Target prediction

Targets for each set of miRNAs were searched in viral genomes using a modified version of miRanda (v September 2008) [29]. This software uses a scoring system based on the complementarities of nucleotides, similar to the Smith-Waterman algorithm. The scoring matrix used for this analysis also allows G = U 'wobble' pairs, which are important for the accurate detection of RNA:RNA duplexes. The algorithm uses folding routines from the Vienna 1.3 RNA secondary structure programming library [61]. Although miRanda was originally designed to search for miRNA targets in animals, it is versatile enough to be modified and has been used to search for targets in viruses and plants, and has proven to be an efficient method [30,62]. The miRanda screenings were repeated several times using randomly generated subsets of either the miRNA or the viral genome sets.

MiRanda screenings were made using different combinations of miRNAs and viral genomes. The main one was plant miRNAs against plant viruses' genomes. This was compared with four other control screenings: (*i*) animal miRNAs vs plant viruses, (*ii*) random 21 nt sequences (*Rand1*) vs plant viruses (*iii*) randomized plant miRNAs (*Rand2*) vs plant viruses and (*iv*) plant miRNAs vs animal-infecting viruses. As a positive control, the plant miRNAs were screened against 190 sequences corresponding to verified miRNA targets in the plant genomes.

The criteria to consider a sequence as a putative miRNA target were: four or fewer mismatches overall, only one or none mismatches in the 5' region of the miRNA (positions 1 to 12), no more than two consecutives mismatches in positions 13 to 21, no mismatches in positions 10 and 11. Additionally, the miRNA:target pair should have low free-energy of bonding (maximum -20 kcal/mol). These criteria are based on experimental work and have been extensively used for miRNA target prediction in various plants [23,63,64].

Four miRanda parameters obtained in the different screenings were used to compare and validate the predicted targets. These parameters were: a) the free-folding energy of the miRNA:target pair, which is commonly used as a measure for miRNA target prediction and indicates the stability of the miRNA:target duplex and the likeliness of correct matching and cleavage; b) the percentage identity, which indicates how many bases are complementary between the miRNAs and the target; c) the Z-score, which is based on a distribution of the shuffled alignment score; a high Z-score means that the alignment is least likely to be the result of chance; and d) the miRanda score, which weights all the others parameters and also each base pair in the alignment based on complementarity and position; it represents a measure of the number of mismatches and their distribution (mismatches in the 5' end of the target are given a higher penalization) [29].

### Statistical analyses

The main variable used to compare the screenings was the matching percentage = [Number of candidates/(Size of the virus' genome (kb) × Number of miRNAs)] × 100, which is the percentage of the screened sample that resulted in target candidates. For statistical analysis, the Shapiro Normality test and Wilcoxon tests were performed with the software R [65].

To compare the targeted regions in the viral genomes, the number of hits in each region was divided by the average size in kilobases of this region in the various viruses' genomes.

## Authors' contributions

ALPQ; designed the experiments, analyzed and organized the data and drafted the manuscript, RN; did the first target predictions experiments, AZ; optimized the software for target prediction and collaborated in data analyses, CL; coordinated the investigation and helped to draft the manuscript. All authors have read and approved the final manuscript.
